# Adjustment Capacity of Maritime Pine Cambial Activity in Drought-Prone Environments

**DOI:** 10.1371/journal.pone.0126223

**Published:** 2015-05-11

**Authors:** Joana Vieira, Filipe Campelo, Sergio Rossi, Ana Carvalho, Helena Freitas, Cristina Nabais

**Affiliations:** 1 CFE – Centre for Functional Ecology, Department of Life Sciences, University of Coimbra, Coimbra, Portugal; 2 Département des Sciences Fondamentales, Université du Québec à Chicoutimi, Chicoutimi, Canada; 3 Key Laboratory of Vegetation Restoration and Management of Degraded Ecosystems, Provincial Key Laboratory of Applied Botany South China Botanical Garden, Chinese Academy of Sciences, Guangzhou, China; INRA - University of Bordeaux, FRANCE

## Abstract

Intra-annual density fluctuations (IADFs) are anatomical features formed in response to changes in the environmental conditions within the growing season. These anatomical features are commonly observed in Mediterranean pines, being more frequent in younger and wider tree rings. However, the process behind IADF formation is still unknown. Weekly monitoring of cambial activity and wood formation would fill this void. Although studies describing cambial activity and wood formation have become frequent, this knowledge is still fragmentary in the Mediterranean region. Here we present data from the monitoring of cambial activity and wood formation in two diameter classes of maritime pine (*Pinus pinaster* Ait.), over two years, in order to test: (i) whether the differences in stem diameter in an even-aged stand were due to timings and/or rates of xylogenesis; (ii) if IADFs were more common in large trees; and (iii) if their formation is triggered by cambial resumption after the summer drought. Larger trees showed higher rates of cell production and longer growing seasons, due to an earlier start and later end of xylogenesis. When a drier winter occurs, larger trees were more affected, probably limiting xylogenesis in the summer months. In both diameter classes a latewood IADF was formed in 2012 in response to late-September precipitation, confirming that the timing of the precipitation event after the summer drought is crucial in determining the resumption of cambial activity and whether or not an IADF is formed. It was the first time that the formation of a latewood IADF was monitored at a weekly time scale in maritime pine. The capacity of maritime pine to adjust cambial activity to the current environmental conditions represents a valuable strategy under the future climate change conditions.

## Introduction

As trees get older and/or taller, physiological processes such as hydraulic conductivity change [1], with consequences in secondary growth. A study comparing xylogenesis in timberline species of different age revealed that old trees (> 250 yr.) had a shorter and delayed period of cambial activity than younger trees (< 80 yr.) [2]. However older trees were also larger, so the effect of age was not completely disentagled from size. In order to isolate the effect of size from xylem formation, Rathgeber and co-authors studied a plantation of *Abies alba* Mill. with trees of different size and social status but similar age [3]. They determined that differences in tree size were due to a higher rate of cell production in dominant trees. In maritime pine (*Pinus pinaster* Ait.) it was also found that in trees with similar size and age the rate of cell production was responsible for the different tree-ring widths observed in the last 15 years [4]. Additionally, the timings of xylogenesis were also linked with the rate of cell production, with a higher rate of cell division being responsible for a later end of xylogenesis [5,6].

Most studies comparing wood formation in trees of different sizes and ages were carried out in boreal or temperate environments, where temperature is the main factor limiting tree growth [7]. Fewer studies have been performed in water limited environments, such as the Mediterranean region, with a climate characterized by mild winters and summer drougth, but also showing a high year-to-year climatic variability, especially in the seasonal distribution of precipitation [8,9]. These climatic features induce a bimodal growth pattern in trees characterized by the presence of two growth periods, one in spring and a second one, less prominent, after the summer drought [10,11]. This pattern can leave its mark in tree rings by triggering the formation of false rings or latewood intra-annual density fluctuations (IADFs) [12].

IADFs are anatomical features formed in response to variations in wheather conditions during the growing season [13–15], and are characterized by the presence of latewood-like cells within earlywood, or earlywood-like cells within latewood [16]. Latewood IADFs are the most commonly found on mediterranean pines [17–22], and have been related with a combination of low previous winter and high late summer precipitation events [23–26]. Dendrochronological studies have identify the triggering climatic factors of latewood IADFs formation, however these studies were performed retrospectively using correlations between the IADFs chronologies and monthly climatic variables without considering wood formation at the intra-annual scale. Although the formation of IADFs has been investigated in Mediterranean species such as *Pinus halepensis* Mill. [10,20] and *Juniperus thurifera* L. [10], this knowledge is still fragmentary. In order to have a complete understanding of latewood IADF formation under mediterranean climate, wood monitorization studies are still necessary.

Several questions still remain open regarding the influence of climate on IADFs formation, the origin of the differences in growth diameter among trees of the same age, and whether there is an influence of tree size on IADFs formation. Campelo and co-authors compared the climatic signal of tree-ring width and IADFs in even-aged maritime pines belonging to two size classes and concluded that although there were no differences in the climatic signal of trees with different size, IADFs were more frequent in trees with larger diameter [24]. In order to determine whether is the timing of xylogenesis or the rate of cell production the responsible for the differences in growth diameter in trees, and under which circumstances latewood IADFs are formed, we have monitored cambial activity and wood formation over two years using an even-aged population divided in two size-classes. Our research hypotheses were that i) larger trees present a higher rate of xylem cell production and a later ending of xylogenesis; ii) that the cambium reactivates after the summer drought forming latewood IADFs; and that iii) IADFs are more common in large trees.

## Material and Methods

### Study site and tree selection

The field study was carried out in "Perimetro florestal dunas de Cantanhede" authorized by "Instituto da Conservação da Natureza e das Florestas". This study did not involve endangered or protected species.

**Fig 1:**
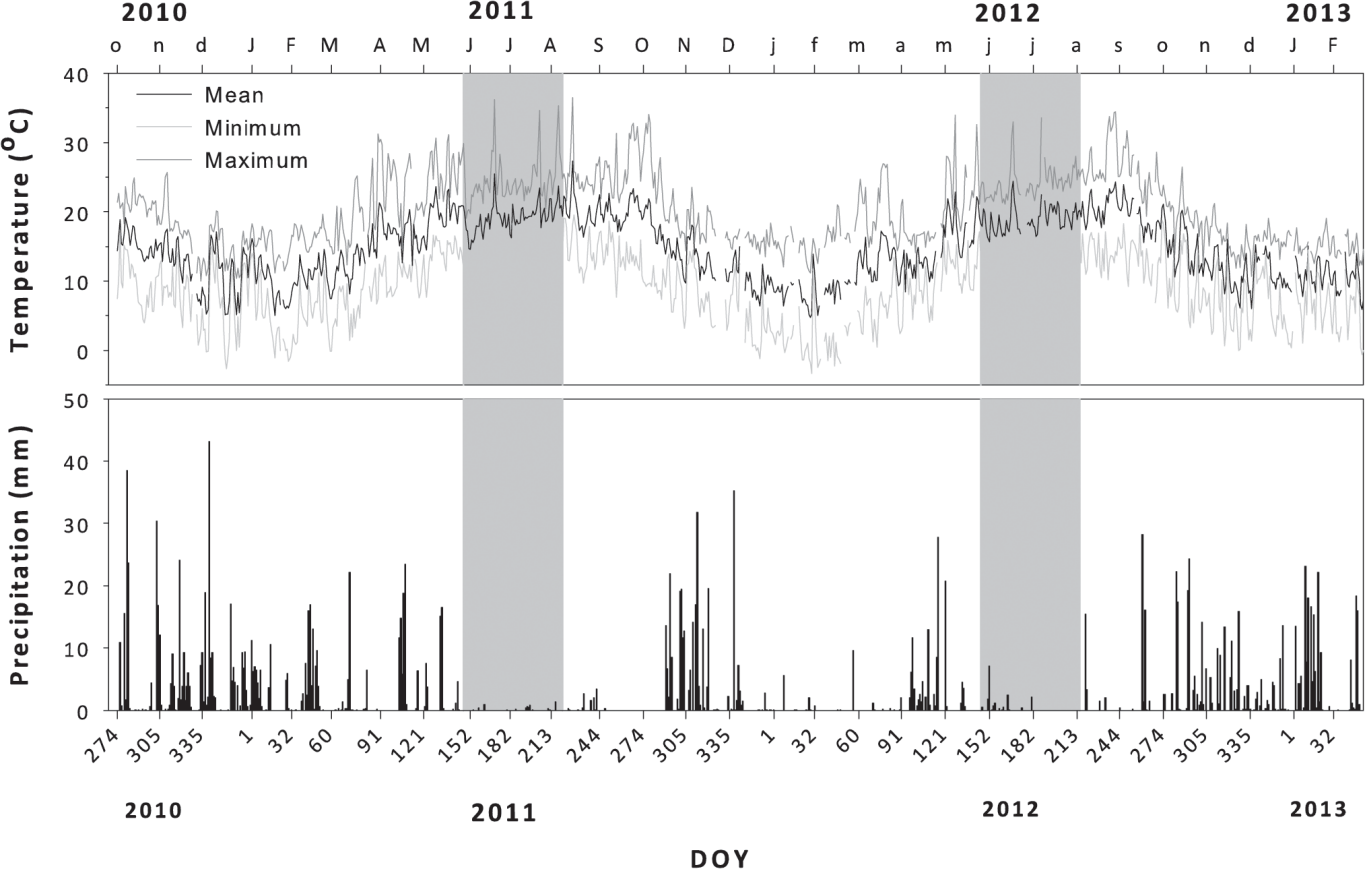
Daily values of temperature and precipitation during 2011 and 2012 in Figueira da Foz, located at 25 km south from the study site (data from Instituto Português de Meteorologia). Grey background area represent periods of drought.

Perimetro Florestal Dunas de Cantanhede is a plantation of maritime pine (*Pinus pinaster* Ait.) on sand dunes, located in the west coast of Portugal (40°21’35.15” N, 8°49’10.06” W; 15 m a.s.l.). The climate is typically Mediterranean with oceanic influence. The mean annual temperature for the last 30 years was 16.1°C, and the total annual precipitation was 965 mm (data downloaded from CRU) [27]. Precipitation occurred mainly in autumn and winter, while the site experienced a pronounced drought in the summer (Fig 1, grey background area). Daily values of maximum and minimum temperature and precipitation for the study period were acquired from the nearest meteorological station (Instituto Português do Mar e da Atmosfera) located in Figueira da Foz, at 25 km south of the study site.

The selected forest stand is a plantation managed by the Portuguese Forest Services, with a stand density of *ca*. 230 trees ha^-1^. The trees selected for this study had an average age at breast height of 47 years and were divided in two groups according to the frequency distribution of stem diameters determined by Campelo and co-authors: large (L-trees; 38.7 ± 3.9 cm) and small trees (S-trees; 23.9 ± 3.0 cm) [24]. Ten dominant trees were selected from each diameter class to monitor the variation in stem diameter and five of them to monitor cambial activity from March 2011 to February 2013. Height, age and diameter at breast height (DBH) from the two diameter classes were compared using a t-test (Table 1).

**Table 1:** Average diameter at breast height (DBH), height and age of small (S) and large (L) trees (± standard deviation) and t-test comparing both diameter classes (n = 5).

Class	S-trees	L-trees	t-test	*p*
Diameter (cm)	22.5 ± 1.9	37.9 ± 2.6	10.58	< 0.001
Height (m)	14.8 ± 0.8	17.0 ± 1.2	3.41	0.009
Age (years)	45.6 ± 4.9	48.8 ± 4.4	1.08	0.312

### Xylem development

Sampling was performed on five trees per class from March 2011 to February 2013 by weekly collecting microcores on the stem using a Trephor [28]. The microcores were collected from 45 cm below and above breast height, in a spiral pattern on the south-facing side of the tree stem in order to minimize the growth variability around the stem [29]. Before sampling, bark was removed in order to reach the living tissues. Between two consecutive sampling dates, microcores were collected at least 5 cm apart to prevent getting resin ducts from adjacent sampling points. The microcores were placed in eppendorfs filled with alcohol (50% in water) and stored at 5 °C to avoid tissue deterioration. In the laboratory, the microcores were dehydrated through successive immersions in alcohol and D-limonene and embedded in paraffin [28]. Transverse sections 6–10 μm thick were cut from the samples with a rotary microtome, stained with cresyl violet acetate (0.17% in water), and immediately observed with a microscope (400–500 x magnification), under visible and polarized light to distinguish the developing xylem cells. Cambial and enlarging cells only have primary cell walls, which, unlike secondary walls do not shine under polarized light. Cambial cells are characterized by thin cell walls and small radial diameters while enlarging cells have a diameter at least twice that of a cambial cell. Wall thickening cells shine under polarized light and show a light violet coloration changing to dark violet at the end of maturation. Lignification appears as a color change from violet to blue, starting at the cell corners and middle lamella and spreading centripetally into the secondary walls. When the entire cell wall presents a blue coloration, lignification is complete and tracheids reach maturation [30]. In each sample, the number of cambial and developing xylem cells was counted along three radial rows and then averaged (Table A in S1 File). Generalized Linear Models (GLM) were performed in SAS 9.4 (SAS Institute Inc., Cary, NC) for testing the differences in the number of cambial and developing cells between diameter classes. Tests of simple effects were performed to test the differences for each sampling date using the SLICE option of the GLM procedure in SAS. The number of earlywood and latewood tracheids formed in the end of 2012 (Table B in S1 File) were also compared between diameter classes and years using a 2-way ANOVA.

**Fig 2:**
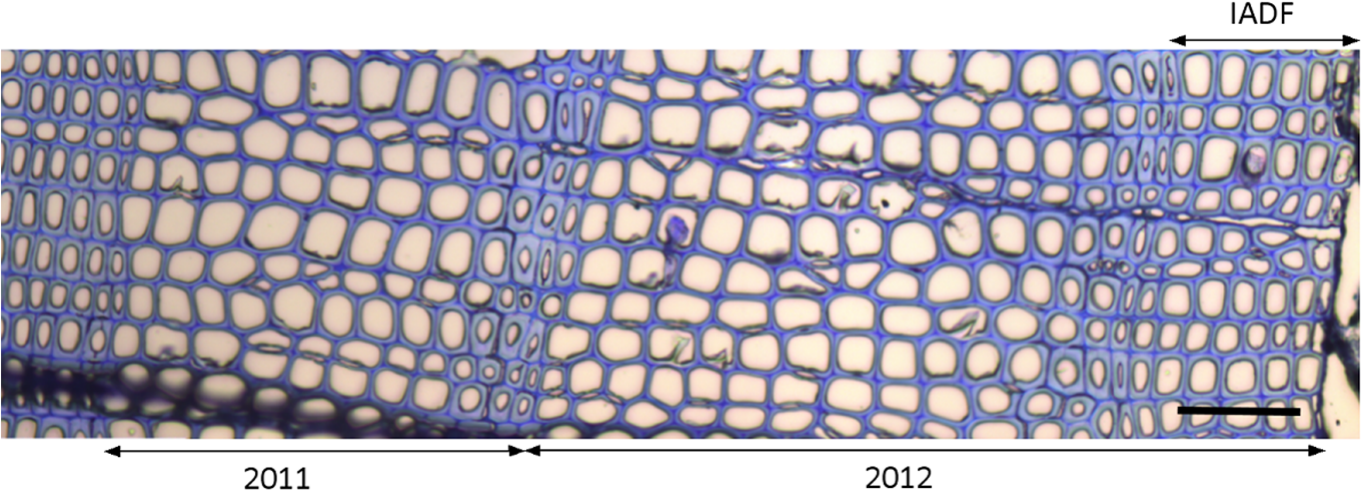
Tree rings formed in 2011 and 2012 (with a latewood IADF). Black line represents 0.1 mm. Picture taken in DOY 37 of 2013 from an S-tree.

The identification of latewood IADFs was made visually in the samples collected in the end of each study year (Fig 2). Latewood IADFs are defined as earlywood-like cells within latewood. According to Mork’s definition latewood tracheids are those in which the width of their common cell walls in the radial direction is equal to or greater than the width of the cell lumen [31]. Thus the earlywood-like tracheids of a latewood IADF present the opposite characteristics in which the lumen radial diameter is wider than that of the common cell walls.

### Band dendrometers

To estimate stem diameter variations, band dendrometers made of astralon (model D1-L, UMS, Munich, Germany) were installed at breast height on 20 trees (10 per diameter class) in January 2011, allowing for a period of adjustment before the beginning of the growing season [32]. Before installation, the superficial section of the bark was carefully removed with a chisel to better adjust the dendrometer to the stem and reduce non-xylematic sources of swelling and shrinking as much as possible [33]. Dendrometers were read weekly to the nearest 0.01 mm (Table C in S1 File). To avoid biases due to the circadian rhythms of water storage and depletion, all measurements were done in the early morning [34].

## Results

### Weather in 2011 and 2012

The two study years presented differences in weather (Fig 1). The average winter temperature (December to February) in 2011 and 2012 was 10.5°C and 9.1°C, respectively. February 2012 was unusually cold, with 15 frozen events and minimum temperatures averaging 0.3°C, while in 2011 the average minimum temperature was 4.7°C. Average spring (March-May) temperatures were 16.1 °C in 2011 and of 14.0°C in 2012. Mean temperatures of 20°C were observed from May to October in 2011, whereas in 2012 temperature was lower, with an average temperature above 20°C being only observed from July to September.

The total annual precipitation was similar among the two years (625 mm in 2011 and 500 mm in 2012), but not its distribution throughout the year (Fig 1). Autumn and winter precipitation prior to growth (October to February) was 560 mm and 296 mm in 2011 and 2012, respectively. After the summer drought, the first rainy events occurred only in mid-October in 2011 whereas in 2012 the first precipitations were observed in mid-September.

### Xylem development and phenology

**Fig 3:**
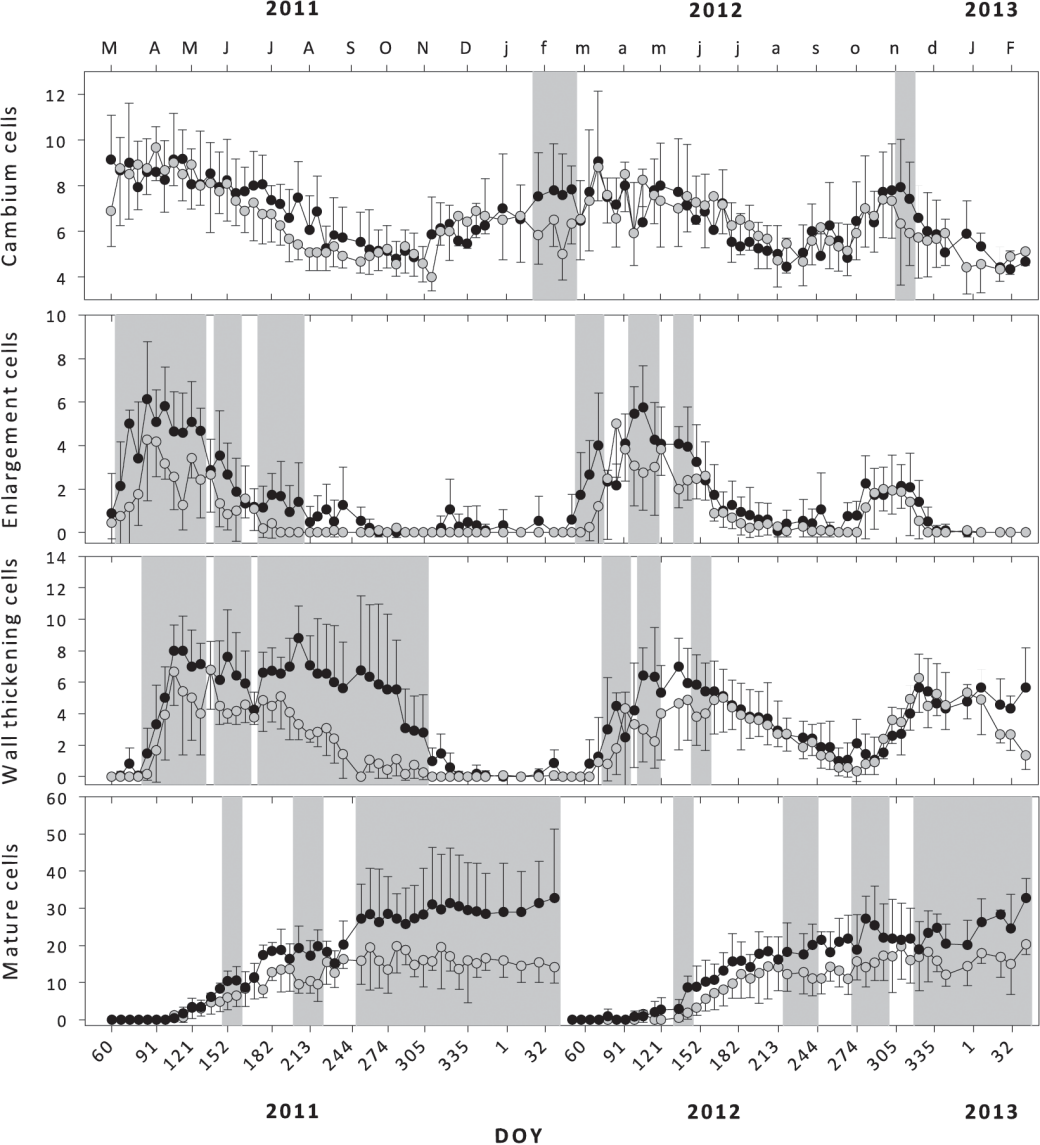
Number of cambial, enlargement, wall thickening and mature cells in large (black dots) and small trees (grey dots). Vertical bars represent standard deviation. The grey background areas represent sampling dates where significant differences in the number of cells were found between diameter classes (test of simple effects in generalized linear models).

Cell division and xylem differentiation phases showed a clear variation in cell number throughout both study years and diameter classes (Fig 3, significant differences highlighted in grey). The seasonal variation in the number of cambial cells was similar in both classes, although L-trees presented a higher number of cells in the first sampling date (March 2011), in February and in November 2012. In 2011 the maximum number of cambial cells occurred between March and June, followed by a continuous decrease until August, time at which the number of cambial cells reached a minimum, lasting until November. In November the number of cambial cells increased again to *ca*. 6 cells remaining constant until mid-January 2012. In the following year (2012) the number of cambial cells presented a similar pattern to the one observed in 2011 until the end of August. In September the number of cambial cells increased again reaching a second maximum in November, with *ca*. 8 cells, decreasing afterwards until a minimum of *ca*. 4 cells in January-February 2012.

In both years, L-trees presented a higher number of enlarging cells from the start of the enlargement phase until the end of July in 2011 (DOY 208) and the end of May in 2012 (DOY 149), time after which the number of enlarging cells was similar between both diameter classes (Fig 3). Cells in enlargement were first observed at the beginning of March in L-trees for both years. In S-trees, this phase started one week later in 2011 and two weeks later in 2012. The enlargement phase of 2011 lasted from March to September in L-trees, and from March to June in S-trees. In 2012, two periods of enlargement were observed in both diameter classes, the first one from March to July and the second one from September to December.

Cell wall deposition also presented differences between diameter classes and years reflecting those observed in the enlargement phase (Fig 3). Significant differences in the number of cells in wall thickening were observed between L- and S- trees from March (DOY 89) to November 2011 (DOY 306) and in March (DOY 86 to 100), April (DOY 114 to 123) and May 2012 (DOY 156 to 163). In 2011 the first cells in wall deposition phase were observed in April (DOY 89), while in 2012 it was at the end of March (DOY 72). The beginning of this phase was delayed in S-trees in both years. In 2011, wall thickening cells were observed in L-trees until the end of November (DOY 327) whereas in S-trees this phase ended in mid-October (DOY 292). In 2012, two periods of cell wall deposition were observed, interrupted by the summer drought. From mid-June to the end of September (DOY 268) both diameter classes presented a similar decrease in the number of cells in wall thickening. In October a minimum number of wall thickening cells was observed, followed by a second period of increase. The second maximum was observed in mid-November (DOY 331) for all trees, followed by a decrease in the number of cells until the end of the monitoring (DOY 49, year 2013). Sampling was interrupted before the end of cell wall deposition.

The first mature cells were observed simultaneously in both diameter classes in 2011 in mid-April (DOY 110), while in 2012 mature cells were first observed in L-trees (DOY 100) and one month later in S-trees (DOY 135). Differences in the number of mature cells were observed between L- and S-trees in June (DOY 152 to 159), August (DOY 208 to 223) and from September (DOY 257) to the end of the study period of 2011. In 2012 those differences were observed in May (DOY 142 to 149), August (DOY 220 to 247), October (DOY 282 to 303) and from November (DOY 331) to end of the monitoring period. At the end of 2011, *ca*. 30 tracheids were produced by L-trees *versus ca*. 15 in S-trees; in 2012 there were also *ca*. 30 tracheids in L-trees versus *ca*. 20 in S-trees (Fig 3).

**Fig 4:**
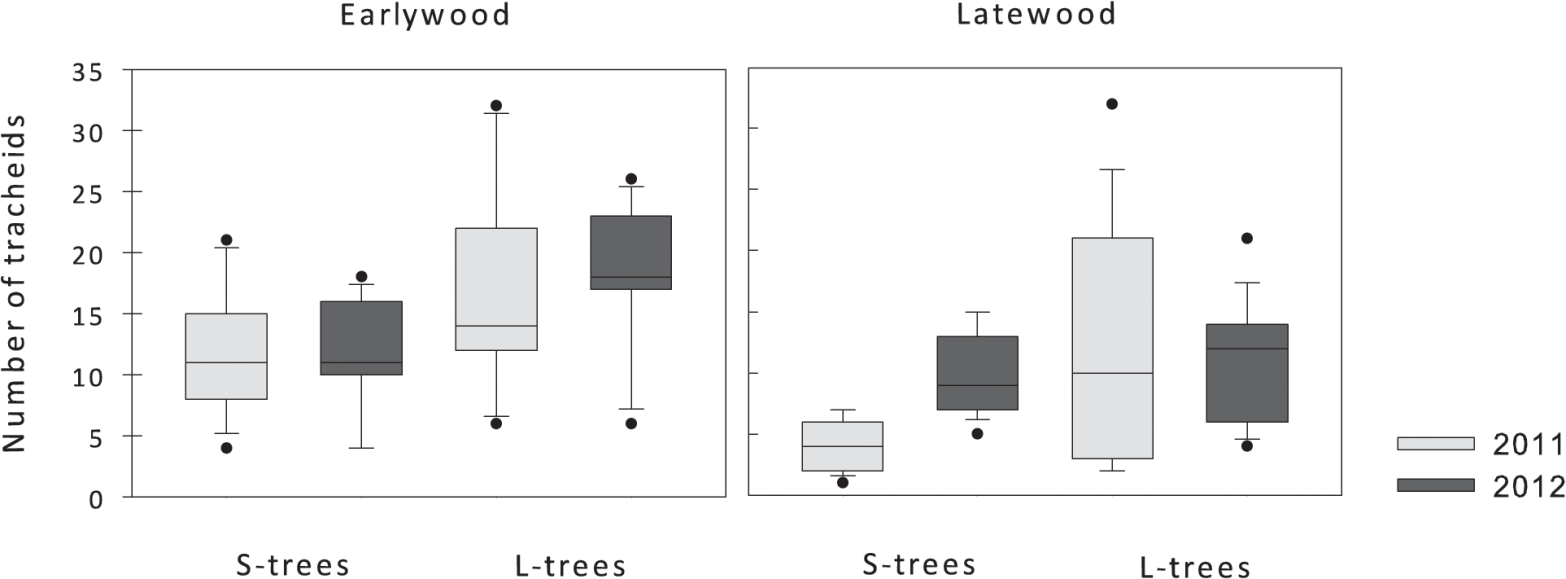
Number of tracheids in earlywood and latewood in 2011 (grey bars) and 2012 (black bars) in large and small trees. Whiskers represent standard deviation, horizontal lines average and the dots outlier values.

Differences in the number of earlywood and latewood tracheids were tested for diameter classes and years (Fig 4). Regarding the number of earlywood tracheids, no significant differences were observed between years (F = 0.016; P = 0.89) but a higher number of earlywood tracheids was observed in L-trees (F = 11.805; P = 0.001). There were also differences in the number of latewood tracheids between size classes (F = 10.132; P = 0.002), with L-trees presenting a higher number of tracheids. No significant differences in the number of latewood tracheids were observed between years (F = 1.896; P = 0.174).

The cells observed in the enlargement phase in September 2012 differentiated in tracheids with a lumen area larger than that of the previously formed latewood, which resulted in the formation of an IADF (Fig 2). The IADF tracheids presented anatomical characteristics similar to those of earlywood tracheids following the definition of Mork, the lumen radial diameter was wider than that of the common cell walls [31]. These structures were observed in 3 L-trees and in 4 S-trees in 2012. There was no IADF formation in 2011 (Fig 2).

### Stem radius variations

**Fig 5:**
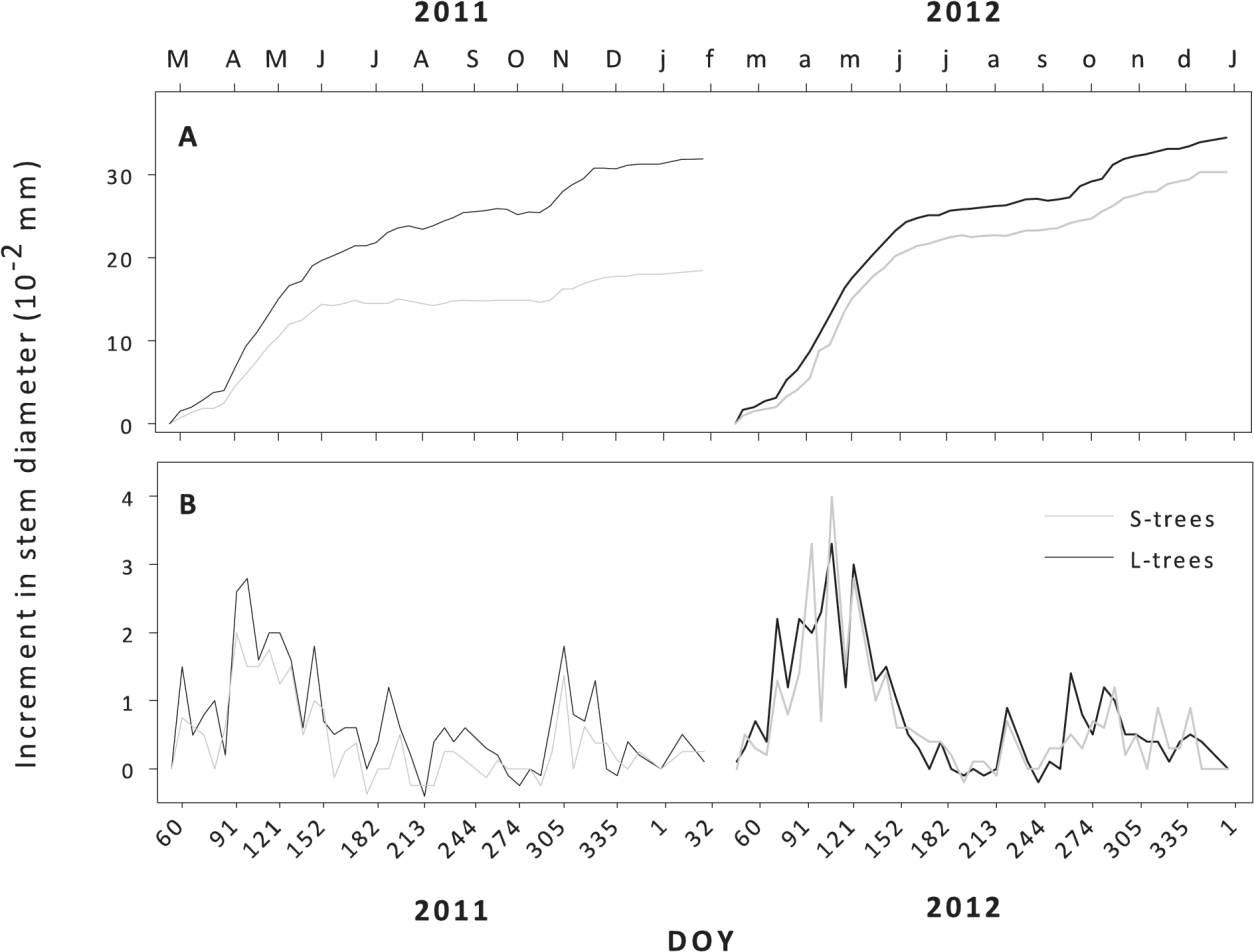
Average cumulative (A) and weekly increments (B) in stem diameter measured with band dendrometers in large (black line) and small trees (grey line).

In both diameter classes and years, the variation in stem diameter showed a clear bimodal pattern, characterized by a pronounced increase in spring, followed by a plateau in late spring and early summer, and a second less marked increase in autumn (Fig 5). The first increment observed in spring occurred during April in 2011 and between mid-March and May in 2012 for both diameter classes (Fig 5B). The autumn increment was more evident in 2011, with weekly increments of 0.18 x 10^–2^ mm being observed in L-trees. The timings were also different between years, the autumn increment period started in November in 2011 and in mid-September in 2012. L-trees presented a higher increase in cumulative stem diameter compared with S-trees in both years (Fig 5A). In 2011 and 2012 L-trees and S-trees showed an increment rate of 3.2 and 3.3 x 10^–2^ mm, and of 1.8 and 3.0 x 10^–2^ mm, respectively.

## Discussion

This study investigated the timings of cambial activity and xylem formation over two years (2011 and 2012) in even-aged maritime pine trees belonging to two diameter classes growing under Mediterranean climate. There were differences in the dynamics of cambial activity between both size-classes and years. A higher number of differentiating xylem cells was observed in L-trees in both years, resulting in wider rings. The differences in the timings of xylogenesis between the two classes were observed in enlargement and cell wall deposition, with both phases starting earlier and presenting a higher number of cells in L-trees. Regarding the two study years, in 2011 L-trees xylogenesis lasted longer than in S-trees. In 2012 September precipitation triggered a second period of cambial activity and xylem formation in October-November, which resulted in the formation of an IADF. These results confirmed the hypotheses that larger trees showed a higher rate of xylem cell production and a longer duration of xylogenesis and that IADFs formation resulted from cambial reactivation.

### Annual dynamics of cambial activity and wood formation

In temperate and cold climates, the vascular cambium presents activity in spring and summer and dormancy in winter. Winter dormancy in trees consists of two phases, the resting and the quiescent phase [35,36]. The resting phase corresponds to the period when cambium is unable to produce new cells, even when supplied with auxin and under favorable conditions and the quiescent state to the period when the cambium is ready to produce new cells [37]. Cambium is reactivated and quiescence broken when the environmental conditions are suitable for growth [38]. The change from a quiescent to an active state is strongly connected with temperature [39–41]. In a previous study on the xylogenesis of maritime pine, Vieira and co-authors determined that cambium cells started to divide and differentiate earlier in years with a warmer winter [42]. The importance of temperature on cambial reactivation has been demonstrated by manipulative experiments demonstrating that heating a portion of the stem triggers an earlier the start of cambial activity [40,43–45]. We observed that the onset of cambial activity, which in this study corresponds to the observation of at least one cell in the enlargement phase in half of the trees, occurred at the same time in both study years (DOY 72, considering all trees). Although the late winter of 2012 was colder when compared to the same period in 2011, the mean annual temperature one week prior to growth onset was above 12°C in both study years, demonstrating the importance of temperature in breaking winter dormancy in the Mediterranean region.

The analysis of the timings of cambial activity in maritime pine revealed two periods of minimal number of cells in the cambium, which indicated a lack of cell production. The first period was during winter although with considerable differences between the studied years. In the winter of 2012, the low number of cambial cells suggests that those trees were dormant. However, the number of cells in the cambium during the winter of 2011 was higher than in 2012, suggesting that the trees can remain quiescent during winter. Studies on the cambial activity of Mediterranean trees have demonstrated that cambium can remain active during mild winters [11,46], especially in evergreen species such as maritime pine. The second period of minimum cambial cells was observed in the summer. This appears to be a defense mechanism against harsh environmental conditions during the summer drought. In a previous study on maritime pine using automatic dendrometers, it was observed that during the summer drought trees were not able to recover the water lost by transpiration during the day, presenting daily negative increments which resulted in the shrinkage of the stem [47]. Thus, in order for maritime pine to maintain needle water potential above the minimal threshold described in the literature [48,49], the water available for the tree is allocated to higher ranked physiological processes such as transpiration, with cambium entering in a quiescent state waiting for more favorable environmental conditions [47,50]. These results indicate that the cambial dynamics of maritime pine, and possibly other evergreen Mediterranean species, present a noteworthy capacity to quickly adjust cambial activity to the current environmental conditions.

Regarding the size classes, xylem differentiation started earlier in L-trees in both study years. Differences in the timings of cambial activity have been observed in trees of different age and social status: cambial activity was reported to start earlier in younger [2] and dominant trees [3]. A possible explanation for the earlier start of enlargement in L-trees is that larger trees may have a better access to resources and a different allocation strategy than S-trees. It was also observed that L-trees presented a longer period of growth and a faster rate of cell production, confirming previous observations by Vieira and co-authors in which fast growing trees presented higher rates of cell production and thus wider rings [4]. In 2011, L-trees presented cells in wall deposition in the summer months whereas the S-trees did not. The capacity of L-trees to prolong xylogenesis might be due to a deeper rooting system or a higher amount of storage water [1,51]. Phillips and co-authors showed that taller trees used a higher percentage of stored water to support daily water transport, which consequently increased photosynthesis on a daily basis [51]. Although L-trees prolonged xylogenesis in the summer months during 2011, in the following year no differences between L- and S-trees were found. This lack of differences might be explained by the meteorological data. In the winter of 2012, previous to the growing season, only half of the precipitation was registered when compared to the same period of 2011. A drier winter might have not completely replenished the soil water reserves which L-trees probably resort to during the summer, explaining the similarities observed between L- and S-trees in the summer of 2012, as suggested by Campelo and co-authors [24]. We can thus conclude that growth of Mediterranean trees is driven by two environmental factors: temperature, limiting cambium onset, and water availability, affecting the duration of xylogenesis.

### Intra-annual density fluctuation

In both years, the number of cambial cells increased after the summer in response to precipitation, which re-hydrated the stem bringing the tree back to a physiologically active state [47]. However only in 2012 cambial cells differentiated into new tracheids in both size classes, suggesting that IADF formation is also regulated by the timing of the triggering factor [19]. Moreover, in 2012 IADFs were formed in both diameter classes, thus the hypothesis that L-trees are more prone to produce latewood IADFs was not supported by our current findings, suggesting that the differences found between large and small trees by Campelo and co-authors [24] could be mediated by the intensity of the triggering factor. The formation of IADFs has been monitored in Mediterranean species such as *J*. *thurifera* and *P*. *halepensis* [10,52] and although they have been frequently observed in maritime pine [17,19,23,24], it was the first time that the dynamics of its formation was recorded with weekly anatomical observations. The anatomical differences between latewood and IADFs cells is that the ratio of cell wall thickness to lumen diameter is lower on IADFs than it is on latewood, thus IADFs are referred to as earlywood-like cells within latewood [11,18]. In order for cambial cells to expand, the pressure potential of the apoplastic water surrounding the cambial cells needs to be superior to the symplastic pressure potential so that water can enter the expanding cell [53]. The pressure that water exercises on the primary wall and the duration of cell enlargement are responsible for the lumen area of the tracheid [54]. In 2012 both diameter classes presented cells in the enlargement phase after the summer drought. The precipitation that fell in September and October 2012 hydrated the trees and increased the xylem pressure potential so that enlargement could occur [47]. Although in 2011 there was also precipitation after the summer drought, it occurred in November. Our results suggest that the timing of the post-summer precipitation event is crucial in determining whether or not an IADF will be formed, supporting the previous dendrochronological studies in maritime pine which related latewood IADFs formation with September-October precipitation [19,22–24,26,55]. Another factor correlated with the formation of IADFs is a dry previous winter [24], which was also observed in 2012 but not in 2011.

### Increment in diameter

The variation in diameter registered by band dendrometers showed a clear synchrony between diameter classes, with L-trees showing the highest increments in both years. Diameter increment consists of several components, including the daily swelling and shrinking of the elastic tissues of the stem [33,47,56]. The onset and maximum stem diameter increments corresponded to the start and maximum number of cells observed in the enlargement phase, respectively. A similar observation was made in *Pinus sylvestris* L. growing in the Eastern Central Alps by Oberhuber and Gruber [57].

A second period of stem diameter increment was observed after the summer in both years, although the formation of new xylem cells was only observed in 2012. This result confirms that band dendrometers are not accurate enough to distinguish between stem hydration and the formation of new xylem cells [42,58]. The timing at which the second increment period was observed corresponded to the occurrence of precipitation in both years, highlighting the importance of the hydration component in stem diameter variation [47].

Comparing the stem increment and final number of xylem cells it was observed that L-trees presented similar increments in both study years whereas S-trees presented higher increment and a superior number of xylem cells in 2012. The year 2012 was characterized by a drier winter prior to growth, which has been shown to have a negative impact on maritime pine tree-ring width [19,24], however the month of April was wetter. Comparing the cumulative increment curves of S-trees it is possible to observe that in 2012 the spring increment lasted until July, whereas in 2011 it reached a plateau in June. April precipitation probably recharged the soils allowing xylogenesis and stem increment to continue, suggesting that S-trees increment was more dependent on current weather conditions.

## Conclusions

For the first time, cambial activity, wood and IADF formation were monitored in maritime pine trees with similar age but belonging to two diameter classes, growing under Mediterranean climate. The earlier onset of the growing season observed in L-trees suggests that intrinsic factors, such as the access to resources, play an important role in growth onset. The longer duration of xylogenesis observed in L-trees during 2011 was probably due to a better access to water reserves by those trees, allowing higher rates of cell production. The formation of a latewood IADF in 2012 revealed the capacity of maritime pine to resume cambial activity and form new cells after the summer drought, which was highly dependent on the timing of the precipitation event. The plasticity of maritime pine cambial activity reflects an opportunistic strategy to manage with the unpredictable Mediterranean climate, which gains special relevance when facing the predicted climate change scenarios. Climatic projections for the Mediterranean region predict a higher frequency of extreme events (flooding and drought) and an increase in the length and intensity of the summer drought. Thus species capable of adjusting growth to current environmental conditions, with quiescent periods during harsh conditions and growth resumption when favorable environmental conditions return, are more likely to succeed under the future climatic conditions. It is also expected that trees will increase the frequency of IADFs [23], with still unknown implications on hydraulic function and wood quality.

## Supporting Information

S1 File Table A, Number of cambial, enlargement, wall thickening and mature cells ± standard deviation in small and large trees in 2011 and 2012. Data used in Fig 3 **. Table B,** Number of total earlywood and latewood tracheids ± SD formed in 2011 and 2012 in small and large trees. Data used in Fig 4. **Table C,** Cumulative and weekly stem increments measured with band dendrometers in small and large trees in 2011 and 2012. Data used in Fig 5.
(DOCX)
