# Supplementary material for: Adjustment Capacity of Maritime Pine Cambial Activity in Drought-Prone Environments
Source: PLoS One. 2015 May 11;10(5):e0126223. doi: 10.1371/journal.pone.0126223 (PMC4427410; doi:10.1371/journal.pone.0126223)
Supplement: Supplementary file 1 [file pone.0126223.s001.docx]

Table A. Number of cambial, enlargment, lignification and mature cells ± standard deviation in Small and Large trees in 2011 and 2012 (n=5). Data used in Figure 3.

		Small trees				Large trees			
	DOY	Cambium	Enlargment	Lignification	Mature	Cambium	Enlargment	Lignification	Mature
	61	6.5 ± 1.5	0.3 ± 0.7	0.0 ± 0.0	0.0 ± 0.0	9.1 ± 2.0	0.9 ± 1.8	0.0 ± 0.0	0.0 ± 0.0
	68	8.1 ± 2.6	0.6 ± 1.2	0.0 ± 0.0	0.0 ± 0.0	8.7 ± 1.4	2.1 ± 2.0	0.1 ± 0.3	0.0 ± 0.0
	75	8.3 ± 1.8	0.9 ± 2.0	0.0 ± 0.0	0.0 ± 0.0	9.0 ± 2.6	5.0 ± 0.6	0.8 ± 1.0	0.0 ± 0.0
	82	8.3 ± 2.1	1.4 ± 1.5	0.0 ± 0.0	0.0 ± 0.0	7.9 ± 2.0	3.4 ± 2.6	0.1 ± 0.3	0.0 ± 0.0
	89	8.1 ± 1.6	3.4 ± 3.0	0.1 ± 0.4	0.0 ± 0.0	8.6 ± 1.5	6.1 ± 2.6	1.5 ± 1.6	0.0 ± 0.0
	96	9.6 ± 1.4	3.3 ± 2.2	1.3 ± 1.9	0.0 ± 0.0	8.6 ± 2.0	5.1 ± 1.5	3.3 ± 2.5	0.0 ± 0.0
	103	8.7 ± 1.7	2.5 ± 1.5	3.1 ± 3.0	0.0 ± 0.0	8.3 ± 1.7	5.8 ± 1.8	5.0 ± 2.0	0.1 ± 0.3
	110	8.8 ± 0.9	2.3 ± 1.4	5.0 ± 3.5	0.8 ± 1.5	9.1 ± 2.0	4.6 ± 1.8	8.0 ± 1.6	0.4 ± 0.7
	117	8.1 ± 1.4	1.5 ± 1.1	4.3 ± 4.2	0.5 ± 1.2	9.2 ± 1.3	4.6 ± 1.8	8.0 ± 2.2	1.8 ± 1.7
	124	8.2 ± 2.3	2.7 ± 1.6	4.0 ± 3.2	2.5 ± 2.8	8.1 ± 1.5	5.1 ± 1.9	7.0 ± 2.3	3.3 ± 2.5
	131	7.6 ± 1.1	1.9 ± 1.8	3.2 ± 2.9	2.4 ± 2.1	8.1 ± 2.3	4.7 ± 1.0	7.1 ± 1.4	3.5 ± 1.7
	139	8.2 ± 1.9	2.3 ± 1.6	5.6 ± 3.0	3.5 ± 3.4	8.5 ± 1.4	2.9 ± 1.4	6.7 ± 1.9	6.1 ± 1.1
	146	7.5 ± 1.1	1.3 ± 1.0	3.7 ± 2.0	3.9 ± 3.0	7.9 ± 1.5	3.5 ± 2.1	6.1 ± 2.5	8.3 ± 1.6
	152	7.8 ± 1.6	0.8 ± 0.7	3.3 ± 1.5	4.8 ± 3.9	8.3 ± 1.8	2.7 ± 1.4	7.6 ± 3.0	10.4 ± 2.7
	159	7.2 ± 1.0	0.8 ± 1.3	3.9 ± 0.9	5.8 ± 2.9	7.7 ± 1.5	1.9 ± 1.6	6.4 ± 2.7	10.5 ± 3.9
	166	6.8 ± 0.8	1.2 ± 1.0	4.1 ± 1.3	7.3 ± 4.6	7.8 ± 1.1	1.3 ± 1.7	5.9 ± 2.1	8.8 ± 4.2
	173	6.9 ± 1.6	0.9 ± 1.0	3.5 ± 0.7	9.5 ± 6.4	8.0 ± 1.5	1.2 ± 0.8	4.3 ± 1.1	11.3 ± 3.9
	180	6.5 ± 1.3	0.1 ± 0.4	4.1 ± 2.3	10.2 ± 4.8	8.1 ± 1.3	1.1 ± 1.0	6.6 ± 1.3	17.4 ± 2.7
	187	6.4 ± 1.3	0.3 ± 0.6	4.3 ± 1.0	14.5 ± 4.3	7.4 ± 0.6	1.7 ± 1.0	6.7 ± 1.5	18.5 ± 2.3
	194	6.2 ± 0.8	0.0 ± 0.0	4.3 ± 2.5	14.7 ± 5.9	7.2 ± 0.9	1.7 ± 1.6	6.5 ± 1.1	18.8 ± 5.5
	201	5.7 ± 0.9	0.0 ± 0.0	3.4 ± 2.1	15.5 ± 6.5	6.6 ± 1.2	0.9 ± 1.2	7.0 ± 1.8	16.4 ± 4.1
	208	5.5 ± 0.6	0.0 ± 0.0	3.1 ± 1.0	12.4 ± 6.4	7.5 ± 1.6	1.4 ± 1.8	8.8 ± 2.0	19.3 ± 6.0
2011	217	5.0 ± 0.8	0.0 ± 0.0	2.6 ± 0.5	13.2 ± 6.5	6.1 ± 1.5	0.5 ± 0.7	7.1 ± 1.9	17.1 ± 4.2
	223	5.1 ± 0.8	0.0 ± 0.0	2.8 ± 0.9	9.6 ± 4.6	6.9 ± 1.6	0.7 ± 1.0	6.5 ± 3.5	19.7 ± 4.4
	230	5.1 ± 0.8	0.0 ± 0.0	2.7 ± 1.8	18.2 ± 7.8	5.3 ± 1.0	1.1 ± 1.2	6.5 ± 3.1	18.2 ± 2.9
	236	5.3 ± 0.8	0.1 ± 0.3	2.1 ± 1.6	14.5 ± 5.2	5.8 ± 1.6	0.5 ± 1.0	6.0 ± 3.6	15.2 ± 3.5
	243	4.7 ± 0.6	0.0 ± 0.0	1.3 ± 1.4	18.5 ± 5.1	5.7 ± 1.3	1.3 ± 1.8	5.6 ± 3.0	20.2 ± 6.5
	257	4.7 ± 0.5	0.0 ± 0.0	0.0 ± 0.0	15.9 ± 6.4	5.5 ± 1.3	0.5 ± 0.6	6.7 ± 4.8	27.2 ± 9.4
	264	5.0 ± 0.8	0.0 ± 0.0	0.9 ± 0.8	20.5 ± 10.5	5.2 ± 1.7	0.2 ± 0.4	6.3 ± 4.6	28.4 ± 12.4
	271	5.1 ± 0.7	0.1 ± 0.3	0.7 ± 0.9	17.6 ± 7.5	5.1 ± 0.9	0.0 ± 0.0	5.9 ± 5.1	26.3 ± 14.3
	278	5.3 ± 0.8	0.0 ± 0.0	0.3 ± 0.5	15.9 ± 7.0	5.1 ± 1.1	0.0 ± 0.0	5.5 ± 4.8	28.5 ± 10.0
	285	4.6 ± 0.5	0.2 ± 0.4	1.1 ± 1.1	19.8 ± 7.2	4.8 ± 0.8	0.0 ± 0.0	5.5 ± 3.1	27.2 ± 6.7
	292	5.4 ± 1.1	0.0 ± 0.0	0.1 ± 0.4	19.1 ± 3.7	5.1 ± 0.9	0.0 ± 0.0	3.1 ± 2.5	25.7 ± 9.9
	299	5.3 ± 1.1	0.0 ± 0.0	0.6 ± 0.8	15.9 ± 3.8	4.9 ± 1.1	0.0 ± 0.0	2.9 ± 2.2	27.3 ± 8.9
	306	4.5 ± 0.7	0.0 ± 0.0	0.3 ± 0.5	18.9 ± 8.7	4.6 ± 0.7	0.0 ± 0.0	2.8 ± 2.4	28.3 ± 12.5
	313	4.0 ± 0.5	0.0 ± 0.0	0.1 ± 0.3	18.9 ± 7.1	5.9 ± 1.6	0.0 ± 0.0	1.0 ± 0.8	31.1 ± 15.3
	320	5.7 ± 0.8	0.0 ± 0.0	0.1 ± 0.3	21.7 ± 11.0	6.1 ± 1.1	0.2 ± 0.6	1.5 ± 1.2	29.6 ± 14.9
	327	5.7 ± 1.3	0.3 ± 0.6	0.2 ± 0.4	20.1 ± 7.2	6.3 ± 0.8	1.1 ± 1.4	0.6 ± 0.9	31.3 ± 14.9
	334	6.4 ± 1.3	0.4 ± 0.9	0.0 ± 0.0	17.1 ± 8.6	5.6 ± 1.0	0.3 ± 0.6	0.1 ± 0.3	30.5 ± 13.8
	341	6.0 ± 1.4	0.1 ± 0.5	0.2 ± 0.4	19.4 ± 12.4	5.5 ± 1.1	0.5 ± 0.7	0.0 ± 0.0	29.5 ± 12.8
	348	6.3 ± 1.4	0.7 ± 0.9	0.3 ± 0.7	19.2 ± 8.1	6.1 ± 1.2	0.3 ± 0.9	0.2 ± 0.6	29.1 ± 13.0
	355	6.5 ± 0.8	0.4 ± 0.7	0.3 ± 0.6	17.6 ± 3.7	6.3 ± 2.1	0.1 ± 0.3	0.1 ± 0.3	28.5 ± 10.9
	369	6.5 ± 1.6	0.5 ± 1.1	1.3 ± 1.6	16.3 ± 4.2	7.0 ± 2.4	0.3 ± 0.7	0.0 ± 0.0	29.0 ± 13.1
	383	6.5 ± 1.8	0.3 ± 0.7	1.8 ± 2.0	15.1 ± 4.1	6.5 ± 1.5	0.0 ± 0.0	0.0 ± 0.0	29.0 ± 10.8
	397	5.7 ± 1.2	0.2 ± 0.4	0.6 ± 0.8	17.4 ± 6.2	7.5 ± 1.9	0.5 ± 1.1	0.1 ± 0.4	31.3 ± 11.2

		Small trees				Large trees			
	**DOY**	**Cambium**	**Enlargment**	**Lignification**	**Mature**	**Cambium**	**Enlargment**	**Lignification**	**Mature**
	58	6.3 ± 0.9	0.0 ± 0.0	0.0 ± 0.0	0.0 ± 0.0	7.8 ± 1.0	0.6 ± 1.2	0.0 ± 0.0	0.0 ± 0.0
	65	6.5 ± 1.8	0.0 ± 0.0	0.0 ± 0.0	0.0 ± 0.0	6.5 ± 1.8	1.7 ± 1.9	0.0 ± 0.0	0.0 ± 0.0
	72	7.3 ± 2.2	0.3 ± 0.6	0.0 ± 0.0	0.0 ± 0.0	7.7 ± 2.7	2.7 ± 1.6	0.8 ± 1.5	0.0 ± 0.0
	79	8.8 ± 1.5	1.2 ± 1.8	0.9 ± 1.9	0.0 ± 0.0	9.1 ± 3.1	4.0 ± 2.4	1.3 ± 2.5	0.9 ± 1.9
	86	7.6 ± 1.2	2.5 ± 2.8	0.8 ± 1.1	0.0 ± 0.0	7.5 ± 0.8	2.3 ± 0.5	3.0 ± 3.3	0.0 ± 0.0
	93	6.6 ± 1.2	5.0 ± 2.4	1.8 ± 1.6	0.0 ± 0.0	7.2 ± 1.2	2.2 ± 1.0	4.5 ± 0.8	0.0 ± 0.0
	100	8.5 ± 1.6	3.8 ± 1.5	4.3 ± 4.9	0.0 ± 0.0	8.0 ± 1.0	4.1 ± 1.4	2.5 ± 2.9	0.8 ± 1.5
	107	5.9 ± 1.4	3.1 ± 1.8	3.3 ± 2.7	1.5 ± 2.2	5.9 ± 2.2	5.5 ± 1.2	4.2 ± 3.0	0.9 ± 1.5
	114	8.3 ± 1.2	2.7 ± 1.7	3.0 ± 2.0	0.0 ± 0.0	6.4 ± 2.3	5.7 ± 1.9	6.4 ± 1.8	2.1 ± 2.9
	123	7.6 ± 1.2	3.0 ± 2.2	2.3 ± 1.7	0.0 ± 0.0	7.8 ± 1.4	4.3 ± 1.7	6.3 ± 3.1	2.7 ± 3.1
	128	7.3 ± 2.0	3.8 ± 1.2	4.0 ± 2.9	0.4 ± 0.8	8.0 ± 1.9	4.1 ± 1.7	5.3 ± 1.7	2.9 ± 2.6
	142	7.0 ± 1.7	2.0 ± 0.9	4.7 ± 3.0	1.8 ± 1.7	7.7 ± 2.3	4.1 ± 0.8	7.0 ± 1.8	8.7 ± 3.0
	149	7.5 ± 1.6	2.4 ± 0.9	4.9 ± 2.6	3.2 ± 2.1	7.1 ± 2.7	3.9 ± 1.8	5.9 ± 2.2	8.8 ± 5.7
	156	7.3 ± 1.0	2.5 ± 0.9	3.8 ± 1.8	5.7 ± 3.3	6.5 ± 1.9	3.3 ± 1.7	5.8 ± 1.9	10.2 ± 5.8
	163	7.1 ± 1.8	2.6 ± 1.1	4.0 ± 2.3	7.1 ± 4.5	6.9 ± 1.6	2.4 ± 1.8	5.4 ± 1.8	10.7 ± 4.3
	170	7.5 ± 1.5	0.9 ± 0.4	4.9 ± 2.1	8.1 ± 4.3	6.1 ± 1.3	1.7 ± 1.4	5.4 ± 2.0	13.2 ± 4.8
	177	7.2 ± 1.3	0.9 ± 0.7	5.0 ± 2.2	9.7 ± 3.9	7.2 ± 1.5	1.0 ± 0.9	5.1 ± 1.7	15.7 ± 7.6
	184	6.3 ± 1.6	0.7 ± 0.5	4.4 ± 1.4	12.2 ± 6.4	5.5 ± 1.7	1.3 ± 1.1	4.5 ± 1.6	15.9 ± 5.1
	191	6.5 ± 1.5	0.4 ± 0.5	3.9 ± 2.2	11.1 ± 5.2	5.3 ± 1.4	0.9 ± 0.7	4.3 ± 1.7	14.1 ± 3.5
	198	6.3 ± 1.1	0.2 ± 0.4	3.7 ± 1.2	12.5 ± 8.1	5.5 ± 1.6	0.8 ± 1.3	3.8 ± 1.7	17.7 ± 6.1
	205	5.8 ± 1.4	0.3 ± 0.5	3.6 ± 1.1	14.3 ± 8.7	5.3 ± 1.1	0.6 ± 0.8	3.8 ± 1.7	18.4 ± 3.9
**2012**	212	5.7 ± 1.3	0.4 ± 0.5	3.3 ± 1.2	14.1 ± 6.3	5.1 ± 1.1	0.6 ± 0.7	3.7 ± 2.3	16.2 ± 6.1
	220	4.7 ± 1.2	0.3 ± 0.5	2.7 ± 0.8	12.2 ± 4.6	5.0 ± 1.3	0.1 ± 0.3	2.9 ± 1.9	18.3 ± 7.8
	227	5.5 ± 1.3	0.1 ± 0.3	2.7 ± 1.1	12.7 ± 5.8	4.5 ± 1.2	0.4 ± 0.6	2.7 ± 1.0	17.5 ± 5.7
	240	4.7 ± 1.0	0.3 ± 0.5	1.9 ± 0.7	11.1 ± 4.6	5.1 ± 1.2	0.5 ± 1.0	2.5 ± 1.1	20.1 ± 5.7
	247	5.6 ± 1.1	0.1 ± 0.3	2.2 ± 0.9	11.1 ± 4.1	6.0 ± 1.2	0.4 ± 0.7	2.4 ± 0.9	21.5 ± 2.2
	254	6.2 ± 1.3	0.1 ± 0.3	1.3 ± 0.8	14.3 ± 3.8	4.9 ± 1.0	1.1 ± 1.7	1.9 ± 1.6	18.1 ± 5.6
	261	5.6 ± 1.2	0.1 ± 0.3	1.3 ± 0.8	13.2 ± 4.4	6.3 ± 1.9	0.1 ± 0.4	1.9 ± 1.6	21.0 ± 6.2
	268	5.3 ± 1.1	0.0 ± 0.0	0.6 ± 0.5	11.0 ± 4.1	5.6 ± 0.8	0.0 ± 0.0	1.0 ± 0.9	21.8 ± 6.3
	275	5.1 ± 1.1	0.0 ± 0.0	0.6 ± 0.6	15.7 ± 5.0	4.8 ± 1.5	0.8 ± 0.6	1.1 ± 1.1	18.8 ± 9.4
	282	5.9 ± 1.2	0.0 ± 0.0	0.3 ± 0.6	14.1 ± 5.3	6.4 ± 1.7	0.8 ± 0.7	2.1 ± 1.5	27.2 ± 6.0
	289	7.0 ± 1.7	1.1 ± 1.4	0.8 ± 0.7	15.3 ± 4.7	7.0 ± 2.3	2.3 ± 1.3	1.4 ± 1.3	25.4 ± 10.6
	296	6.7 ± 1.6	1.8 ± 1.7	0.9 ± 0.8	17.2 ± 6.7	6.4 ± 1.4	1.7 ± 1.2	1.1 ± 1.4	22.1 ± 9.2
	303	7.4 ± 1.7	2.0 ± 1.0	2.4 ± 1.2	17.0 ± 9.8	7.7 ± 1.8	1.7 ± 1.3	1.5 ± 0.8	21.9 ± 10.4
	310	7.3 ± 1.6	1.9 ± 1.1	3.6 ± 1.5	19.8 ± 8.9	7.8 ± 2.0	1.9 ± 1.6	2.6 ± 1.8	21.5 ± 10.0
	317	6.3 ± 2.7	1.9 ± 0.7	3.5 ± 2.1	16.0 ± 6.1	7.9 ± 2.1	2.1 ± 1.0	2.7 ± 1.6	21.8 ± 8.2
	324	5.9 ± 1.4	1.4 ± 1.5	4.9 ± 2.0	16.9 ± 9.0	7.4 ± 1.6	2.1 ± 1.6	4.0 ± 1.8	19.0 ± 7.3
	331	5.7 ± 1.8	0.5 ± 0.8	6.3 ± 2.2	18.3 ± 8.1	6.6 ± 1.4	1.4 ± 1.0	5.7 ± 2.1	23.4 ± 5.9
	338	5.6 ± 1.6	0.0 ± 0.0	4.5 ± 1.4	15.8 ± 2.9	6.0 ± 1.7	0.5 ± 0.7	5.4 ± 2.1	24.8 ± 3.7
	345	5.7 ± 1.2	0.0 ± 0.0	5.3 ± 1.4	12.1 ± 3.1	5.9 ± 1.5	0.1 ± 0.3	4.7 ± 2.5	20.5 ± 5.3
	352	5.9 ± 1.4	0.0 ± 0.0	4.5 ± 1.6	14.4 ± 5.3	5.1 ± 1.4	0.1 ± 0.3	4.3 ± 2.3	20.2 ± 6.6
	369	4.4 ± 1.2	0.1 ± 0.3	5.3 ± 1.7	17.9 ± 2.8	5.9 ± 1.5	0.0 ± 0.0	4.8 ± 1.1	26.3 ± 6.2
	380	4.6 ± 1.2	0.0 ± 0.0	4.9 ± 2.2	16.9 ± 5.4	5.3 ± 0.6	0.0 ± 0.0	5.7 ± 1.2	28.3 ± 1.2
	395	4.3 ± 0.5	0.0 ± 0.0	2.7 ± 0.5	15.0 ± 8.2	4.4 ± 0.9	0.0 ± 0.0	4.6 ± 1.6	24.6 ± 9.1
	403	4.9 ± 0.8	0.0 ± 0.0	2.7 ± 1.0	20.3 ± 2.8	4.3 ± 0.8	0.0 ± 0.0	4.3 ± 0.8	32.7 ± 5.4
	415	5.1 ± 0.6	0.0 ± 0.0	1.3 ± 0.9	20.6 ± 3.3	4.7 ± 0.6	0.0 ± 0.0	5.7 ± 2.5	27.7 ± 1.2

Table B. Number of total earlywood and latewood tracheids ± SD formed in 2011 and 2012 in small and large trees (n=5). Data used in Figure 4.

	**2011**		**2012**	
	**Earlywood**	**Latewood**	**Earlywood**	**Latewood**
**Small trees**	12.3 ± 5.4	4.0 ± 1.9	11.9 ± 5.0	9.9 ± 3.1
**Large trees**	17.1 ± 8.0	12.6 ± 9.8	17.9 ± 6.2	10.3 ± 4.2

Table C. Cumulative and weekly stem increments measured with band dendrometers in small and large trees in 2011 and 2012 (n=5). Data used in Figure 5.

		Small trees		Large trees	
	DOY	Cumulative	Weekly	Cumulative	Weekly
2011	61	0.011 ± 0.011	0.008 ± 0.011	0.015 ± 0.010	0.02 ± 0.01
	68	0.016 ± 0.008	0.006 ± 0.009	0.020 ± 0.014	0.00 ± 0.00
	75	0.021 ± 0.005	0.005 ± 0.004	0.028 ± 0.018	0.01 ± 0.00
	82	0.022 ± 0.012	0.000 ± 0.008	0.038 ± 0.024	0.01 ± 0.01
	89	0.026 ± 0.015	0.006 ± 0.011	0.040 ± 0.025	0.00 ± 0.00
	96	0.044 ± 0.023	0.020 ± 0.011	0.066 ± 0.031	0.03 ± 0.01
	103	0.057 ± 0.032	0.015 ± 0.014	0.094 ± 0.038	0.03 ± 0.01
	110	0.069 ± 0.043	0.015 ± 0.015	0.110 ± 0.040	0.02 ± 0.01
	117	0.085 ± 0.048	0.017 ± 0.005	0.130 ± 0.045	0.02 ± 0.01
	124	0.094 ± 0.059	0.013 ± 0.013	0.150 ± 0.055	0.02 ± 0.01
	131	0.108 ± 0.064	0.015 ± 0.005	0.166 ± 0.058	0.02 ± 0.01
	139	0.113 ± 0.068	0.005 ± 0.009	0.172 ± 0.058	0.01 ± 0.01
	146	0.121 ± 0.073	0.010 ± 0.008	0.190 ± 0.058	0.02 ± 0.00
	152	0.128 ± 0.075	0.009 ± 0.004	0.197 ± 0.057	0.01 ± 0.00
	159	0.135 ± 0.069	-0.001 ± 0.024	0.202 ± 0.063	0.01 ± 0.01
	166	0.137 ± 0.071	0.002 ± 0.004	0.208 ± 0.064	0.01 ± 0.01
	173	0.141 ± 0.067	0.004 ± 0.004	0.214 ± 0.067	0.01 ± 0.01
	180	0.136 ± 0.072	-0.004 ± 0.007	0.214 ± 0.071	0.00 ± 0.01
	187	0.136 ± 0.072	0.000 ± 0.000	0.218 ± 0.076	0.00 ± 0.01
	194	0.136 ± 0.072	0.000 ± 0.000	0.230 ± 0.083	0.01 ± 0.01
	201	0.140 ± 0.075	0.005 ± 0.005	0.236 ± 0.086	0.01 ± 0.01
	208	0.138 ± 0.075	-0.003 ± 0.004	0.238 ± 0.094	0.00 ± 0.01
	217	0.136 ± 0.072	-0.003 ± 0.004	0.234 ± 0.097	0.00 ± 0.01
	223	0.134 ± 0.070	-0.003 ± 0.004	0.238 ± 0.098	0.00 ± 0.01
	230	0.137 ± 0.071	0.003 ± 0.005	0.244 ± 0.101	0.01 ± 0.00
	236	0.140 ± 0.068	0.003 ± 0.004	0.248 ± 0.105	0.00 ± 0.00
	243	0.141 ± 0.069	0.001 ± 0.002	0.254 ± 0.106	0.01 ± 0.00
	257	0.140 ± 0.068	-0.001 ± 0.002	0.257 ± 0.112	0.00 ± 0.01
	264	0.141 ± 0.069	0.001 ± 0.002	0.259 ± 0.115	0.00 ± 0.00
	271	0.141 ± 0.069	0.000 ± 0.000	0.258 ± 0.115	0.00 ± 0.00
	278	0.149 ± 0.075	0.000 ± 0.000	0.252 ± 0.112	0.00 ± 0.00
	285	0.149 ± 0.075	0.000 ± 0.000	0.255 ± 0.115	0.00 ± 0.00
	292	0.140 ± 0.068	-0.003 ± 0.006	0.254 ± 0.116	0.00 ± 0.00
	299	0.146 ± 0.066	0.003 ± 0.008	0.262 ± 0.119	0.01 ± 0.01
	307	0.164 ± 0.072	0.014 ± 0.016	0.280 ± 0.129	0.02 ± 0.01
	313	0.164 ± 0.072	0.000 ± 0.000	0.288 ± 0.131	0.01 ± 0.00
	320	0.171 ± 0.077	0.006 ± 0.004	0.295 ± 0.129	0.01 ± 0.00
	327	0.174 ± 0.074	0.004 ± 0.004	0.308 ± 0.140	0.01 ± 0.02
	334	0.177 ± 0.070	0.004 ± 0.004	0.308 ± 0.140	0.00 ± 0.00
	341	0.178 ± 0.073	0.001 ± 0.004	0.307 ± 0.139	0.00 ± 0.00
	348	0.178 ± 0.073	0.000 ± 0.000	0.311 ± 0.137	0.00 ± 0.00
	355	0.180 ± 0.073	0.002 ± 0.003	0.313 ± 0.136	0.00 ± 0.00
	369	0.180 ± 0.073	0.000 ± 0.000	0.313 ± 0.134	0.00 ± 0.00
	383	0.182 ± 0.076	0.002 ± 0.004	0.318 ± 0.134	0.01 ± 0.01

		Small trees		Large trees	
	DOY	Cumulative	Weekly	Cumulative	Weekly
	58	0.010 ± 0.012	0.005 ± 0.005	0.017 ± 0.008	0.00 ± 0.00
	65	0.015 ± 0.017	0.003 ± 0.003	0.020 ± 0.012	0.01 ± 0.01
	72	0.018 ± 0.018	0.002 ± 0.004	0.027 ± 0.015	0.00 ± 0.00
	79	0.020 ± 0.022	0.013 ± 0.009	0.031 ± 0.016	0.02 ± 0.01
	86	0.033 ± 0.025	0.008 ± 0.006	0.053 ± 0.006	0.01 ± 0.00
	93	0.041 ± 0.028	0.014 ± 0.007	0.065 ± 0.007	0.02 ± 0.01
	101	0.055 ± 0.032	0.033 ± 0.030	0.087 ± 0.010	0.02 ± 0.01
	107	0.088 ± 0.028	0.007 ± 0.021	0.107 ± 0.021	0.02 ± 0.01
	114	0.095 ± 0.033	0.040 ± 0.019	0.130 ± 0.020	0.03 ± 0.01
	123	0.135 ± 0.042	0.015 ± 0.004	0.163 ± 0.031	0.01 ± 0.00
	128	0.150 ± 0.044	0.028 ± 0.009	0.175 ± 0.027	0.03 ± 0.01
	142	0.178 ± 0.045	0.010 ± 0.006	0.205 ± 0.032	0.01 ± 0.00
	149	0.188 ± 0.048	0.014 ± 0.004	0.218 ± 0.031	0.01 ± 0.01
	156	0.202 ± 0.048	0.006 ± 0.005	0.233 ± 0.028	0.01 ± 0.01
	163	0.208 ± 0.049	0.006 ± 0.009	0.243 ± 0.033	0.01 ± 0.01
	170	0.214 ± 0.050	0.027 ± 0.052	0.248 ± 0.027	0.00 ± 0.00
	177	0.217 ± 0.053	0.004 ± 0.004	0.251 ± 0.029	0.00 ± 0.01
	184	0.221 ± 0.055	0.004 ± 0.002	0.251 ± 0.030	0.00 ± 0.00
	191	0.225 ± 0.056	0.002 ± 0.003	0.257 ± 0.032	0.00 ± 0.00
	199	0.227 ± 0.058	-0.002 ± 0.004	0.258 ± 0.036	0.00 ± 0.00
**2**	205	0.225 ± 0.058	0.001 ± 0.002	0.259 ± 0.041	0.00 ± 0.00
**0**	212	0.226 ± 0.057	0.001 ± 0.002	0.261 ± 0.045	0.00 ± 0.00
**1**	220	0.227 ± 0.058	-0.001 ± 0.002	0.262 ± 0.050	0.00 ± 0.00
**2**	227	0.226 ± 0.057	0.007 ± 0.006	0.263 ± 0.049	0.01 ± 0.01
	240	0.233 ± 0.061	0.000 ± 0.000	0.270 ± 0.046	0.00 ± 0.00
	247	0.233 ± 0.061	0.000 ± 0.000	0.271 ± 0.045	0.00 ± 0.00
	254	0.234 ± 0.060	0.003 ± 0.003	0.269 ± 0.046	0.00 ± 0.00
	261	0.236 ± 0.060	0.003 ± 0.003	0.270 ± 0.046	0.00 ± 0.00
	268	0.241 ± 0.057	0.005 ± 0.004	0.273 ± 0.050	0.01 ± 0.01
	275	0.245 ± 0.058	0.003 ± 0.003	0.286 ± 0.058	0.01 ± 0.01
	282	0.247 ± 0.060	0.007 ± 0.007	0.292 ± 0.059	0.01 ± 0.00
	289	0.256 ± 0.064	0.006 ± 0.007	0.295 ± 0.057	0.01 ± 0.01
	296	0.262 ± 0.066	0.012 ± 0.011	0.312 ± 0.070	0.01 ± 0.01
	303	0.272 ± 0.078	0.002 ± 0.003	0.319 ± 0.066	0.01 ± 0.01
	310	0.275 ± 0.078	0.005 ± 0.004	0.322 ± 0.065	0.01 ± 0.00
	317	0.279 ± 0.081	0.000 ± 0.000	0.325 ± 0.064	0.00 ± 0.00
	324	0.280 ± 0.080	0.009 ± 0.007	0.328 ± 0.062	0.00 ± 0.00
	331	0.289 ± 0.085	0.003 ± 0.003	0.331 ± 0.061	0.00 ± 0.00
	338	0.292 ± 0.086	0.003 ± 0.003	0.331 ± 0.061	0.00 ± 0.00
	345	0.294 ± 0.088	0.009 ± 0.012	0.334 ± 0.060	0.01 ± 0.01
	352	0.303 ± 0.100	0.000 ± 0.000	0.339 ± 0.058	0.00 ± 0.01
	369	0.303 ± 0.100	0.000 ± 0.000	0.345 ± 0.059	0.00 ± 0.00
	380	0.305 ± 0.098	0.015 ± 0.010	0.345 ± 0.059	0.02 ± 0.01
	395	0.318 ± 0.108	0.000 ± 0.000	0.362 ± 0.050	0.00 ± 0.00
